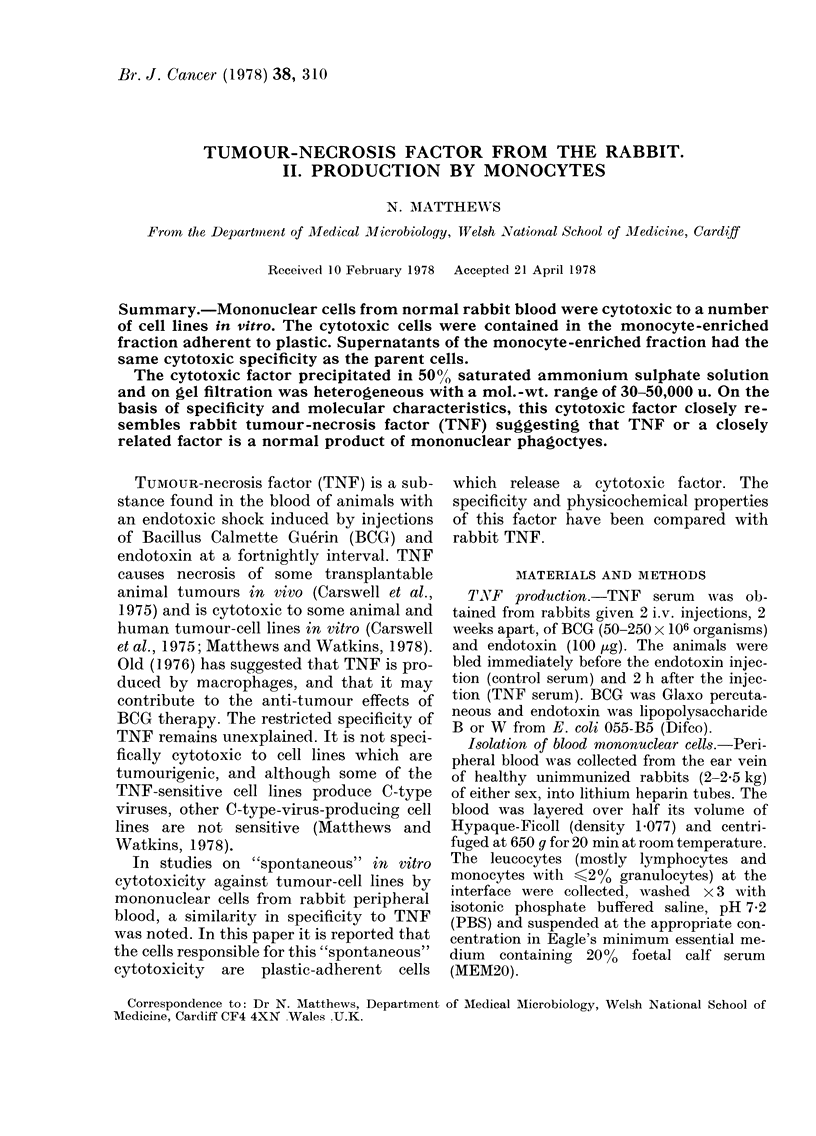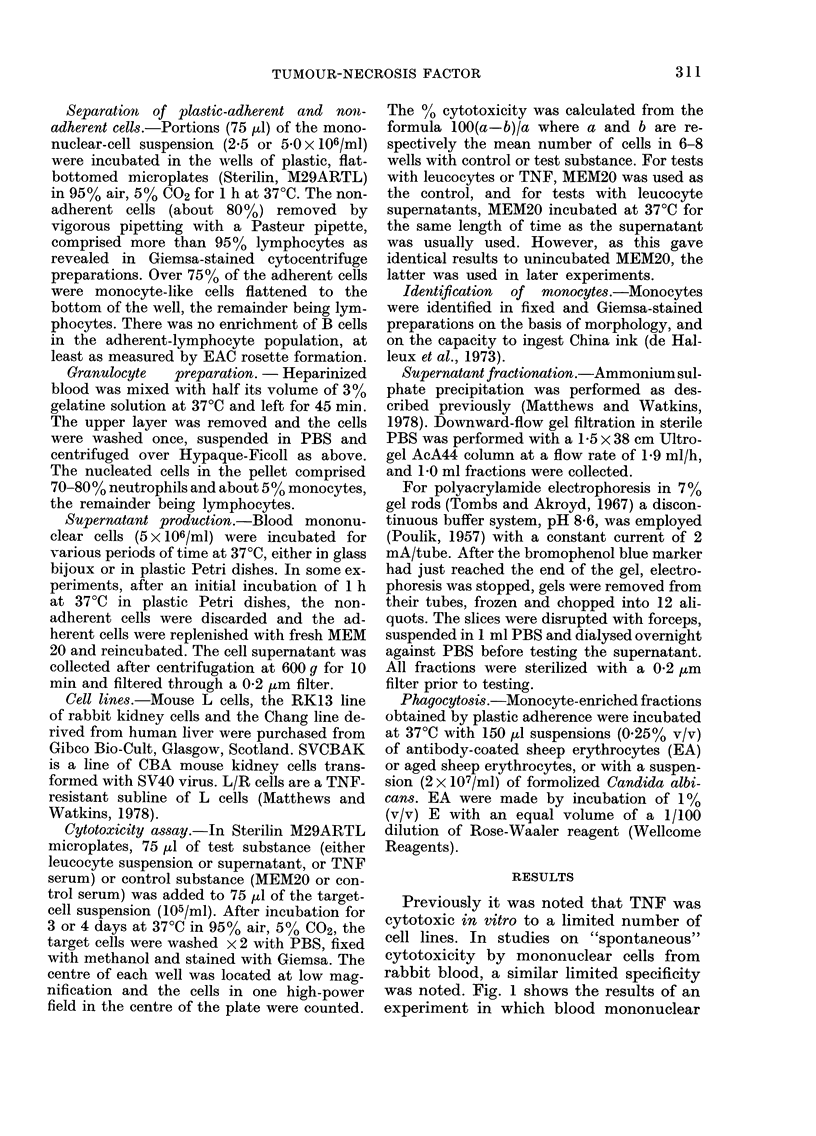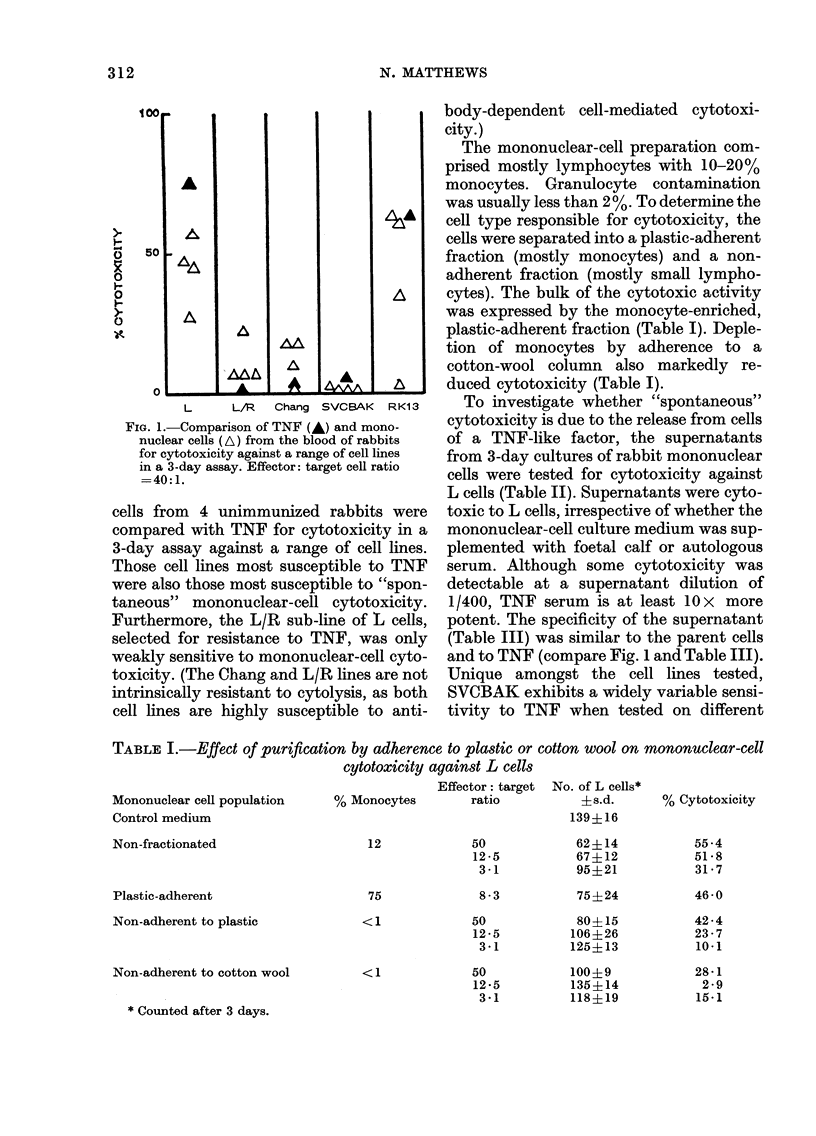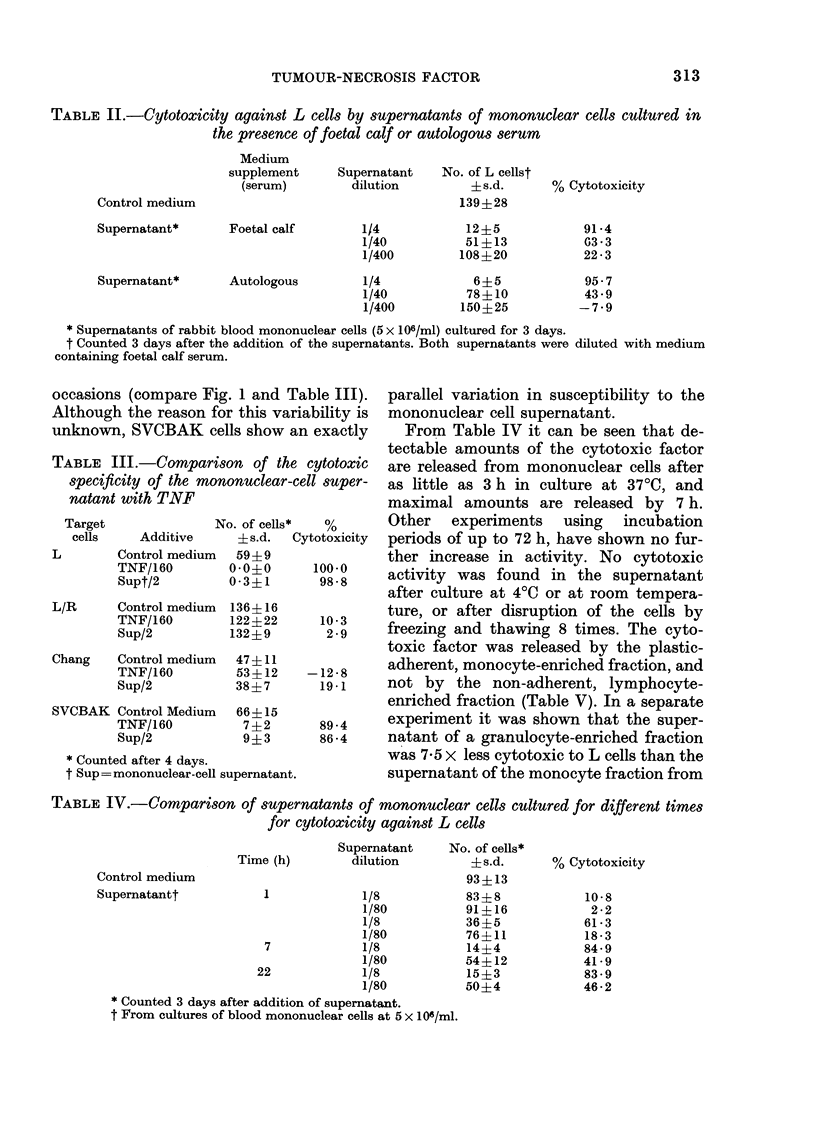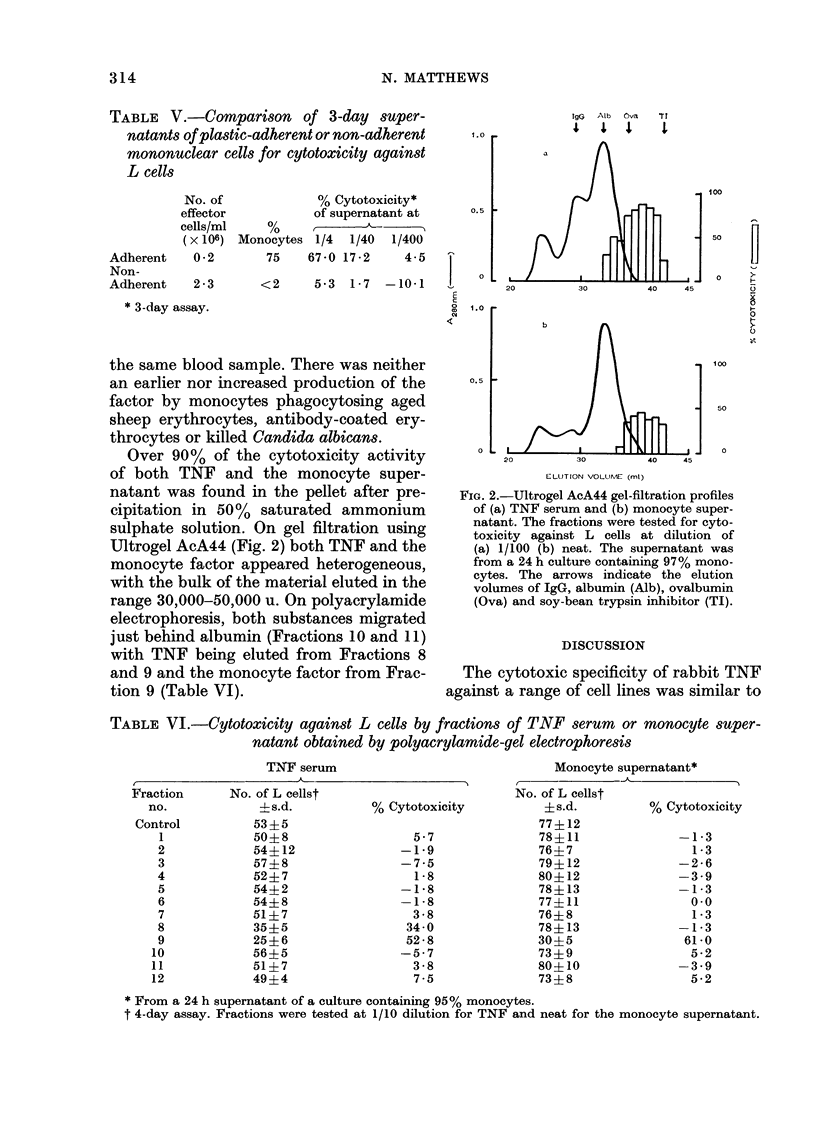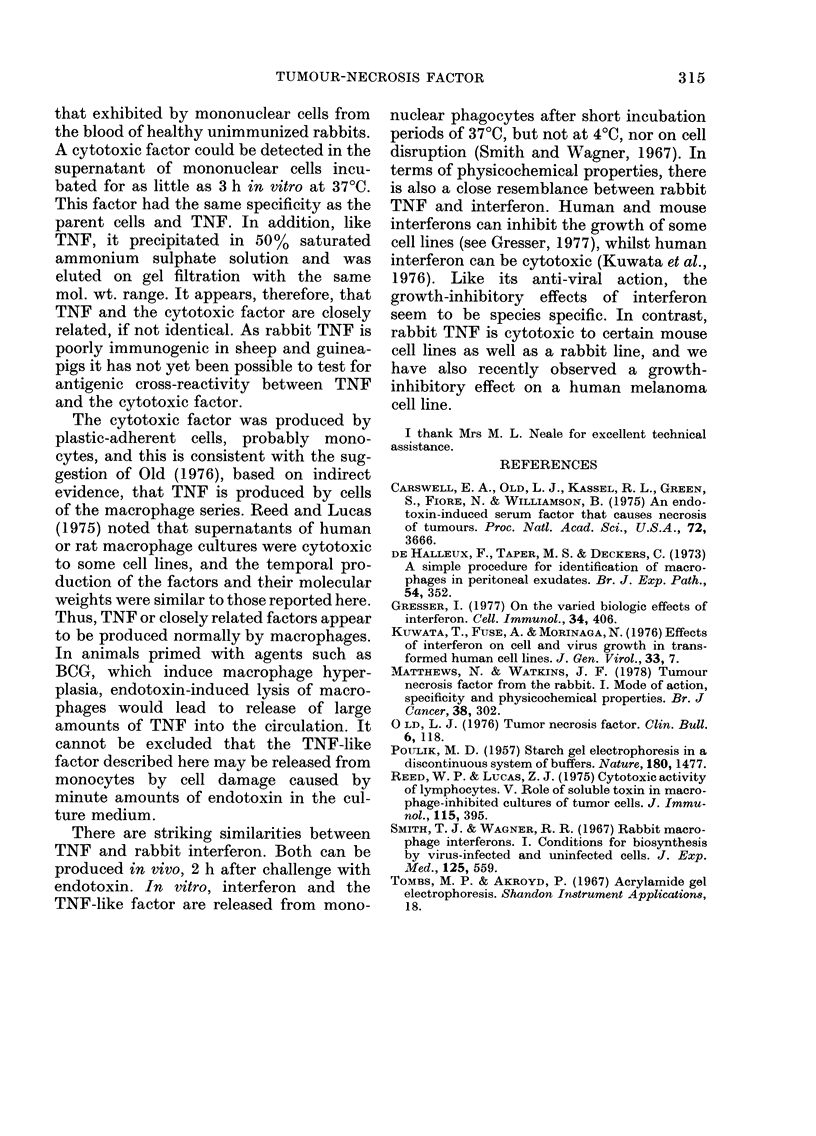# Tumour-necrosis factor from the rabbit. II. Production by monocytes.

**DOI:** 10.1038/bjc.1978.203

**Published:** 1978-08

**Authors:** N. Matthews

## Abstract

Mononuclear cells from normal rabbit blood were cytotoxic to a number of cell lines in vitro. The cytotoxic cells were contained in the monocyte-enriched fraction adherent to plastic. Supernatants of the monocyte-enriched fraction had the same cytotoxic specificity as the parent cells. The cytotoxic factor precipitated in 50% saturated ammonium sulphate solution and on gel filtration was heterogeneous with a mol.-wt. range of 30--50,000 u. On the basis of specificity and molecular characteristics, this cytotoxic factor closely resembles rabbit tumour-necrosis factor (TNF) suggesting that TNF or a closely related factor is a normal product of mononuclear phagocytes.


Br. J. Cancer (1978) 38, 310

### TUMOUR-NECROSIS FACTOR FROM THE RABBIT. II. PRODUCTION BY MONOCYTES

N. MATTHEWS

From the Department of Medical Microbiology, Welsh National School of Medicine, Cardiff

Received 10 February 1978 Accepted 21 April 1978

**Summary.—Mononuclear cells from normal rabbit blood were cytotoxic to a number of cell lines *in vitro*. The cytotoxic cells were contained in the monocyte-enriched fraction adherent to plastic. Supernatants of the monocyte-enriched fraction had the same cytotoxic specificity as the parent cells.**

**The cytotoxic factor precipitated in 50% saturated ammonium sulphate solution and on gel filtration was heterogeneous with a mol.-wt. range of 30–50,000 u. On the basis of specificity and molecular characteristics, this cytotoxic factor closely resembles rabbit tumour-necrosis factor (TNF) suggesting that TNF or a closely related factor is a normal product of mononuclear phagoctyes.**

TUMOUR-necrosis factor (TNF) is a substance found in the blood of animals with an endotoxic shock induced by injections of Bacillus Calmette Guérin (BCG) and endotoxin at a fortnightly interval. TNF causes necrosis of some transplantable animal tumours *in vivo* (Carswell *et al.*, 1975) and is cytotoxic to some animal and human tumour-cell lines *in vitro* (Carswell *et al.*, 1975; Matthews and Watkins, 1978). Old (1976) has suggested that TNF is produced by macrophages, and that it may contribute to the anti-tumour effects of BCG therapy. The restricted specificity of TNF remains unexplained. It is not specifically cytotoxic to cell lines which are tumourigenic, and although some of the TNF-sensitive cell lines produce C-type viruses, other C-type-virus-producing cell lines are not sensitive (Matthews and Watkins, 1978).

In studies on "spontaneous" *in vitro* cytotoxicity against tumour-cell lines by mononuclear cells from rabbit peripheral blood, a similarity in specificity to TNF was noted. In this paper it is reported that the cells responsible for this "spontaneous" cytotoxicity are plastic-adherent cells which release a cytotoxic factor. The specificity and physicochemical properties of this factor have been compared with rabbit TNF.

### MATERIALS AND METHODS

*TNF production.*—TNF serum was obtained from rabbits given 2 i.v. injections, 2 weeks apart, of BCG ($50–250 \times 10^6$ organisms) and endotoxin (100 $\mu$g). The animals were bled immediately before the endotoxin injection (control serum) and 2 h after the injection (TNF serum). BCG was Glaxo percutaneous and endotoxin was lipopolysaccharide B or W from *E. coli* 055-B5 (Difco).

*Isolation of blood mononuclear cells.*—Peripheral blood was collected from the ear vein of healthy unimmunized rabbits (2–2·5 kg) of either sex, into lithium heparin tubes. The blood was layered over half its volume of Hypaque-Ficoll (density 1·077) and centrifuged at 650 *g* for 20 min at room temperature. The leucocytes (mostly lymphocytes and monocytes with $\leqslant 2\%$ granulocytes) at the interface were collected, washed $\times 3$ with isotonic phosphate buffered saline, pH 7·2 (PBS) and suspended at the appropriate concentration in Eagle's minimum essential medium containing 20% foetal calf serum (MEM20).

Correspondence to: Dr N. Matthews, Department of Medical Microbiology, Welsh National School of Medicine, Cardiff CF4 4XN .Wales .U.K.

*Separation of plastic-adherent and non-adherent cells.*—Portions (75 $\mu$l) of the mononuclear-cell suspension (2·5 or $5{\cdot}0 \times 10^6$/ml) were incubated in the wells of plastic, flat-bottomed microplates (Sterilin, M29ARTL) in 95% air, 5% $CO_2$ for 1 h at 37°C. The non-adherent cells (about 80%) removed by vigorous pipetting with a Pasteur pipette, comprised more than 95% lymphocytes as revealed in Giemsa-stained cytocentrifuge preparations. Over 75% of the adherent cells were monocyte-like cells flattened to the bottom of the well, the remainder being lymphocytes. There was no enrichment of B cells in the adherent-lymphocyte population, at least as measured by EAC rosette formation.

*Granulocyte preparation.* — Heparinized blood was mixed with half its volume of 3% gelatine solution at 37°C and left for 45 min. The upper layer was removed and the cells were washed once, suspended in PBS and centrifuged over Hypaque-Ficoll as above. The nucleated cells in the pellet comprised 70–80% neutrophils and about 5% monocytes, the remainder being lymphocytes.

*Supernatant production.*—Blood mononuclear cells ($5 \times 10^6$/ml) were incubated for various periods of time at 37°C, either in glass bijoux or in plastic Petri dishes. In some experiments, after an initial incubation of 1 h at 37°C in plastic Petri dishes, the non-adherent cells were discarded and the adherent cells were replenished with fresh MEM 20 and reincubated. The cell supernatant was collected after centrifugation at 600 *g* for 10 min and filtered through a 0·2 $\mu$m filter.

*Cell lines.*—Mouse L cells, the RK13 line of rabbit kidney cells and the Chang line derived from human liver were purchased from Gibco Bio-Cult, Glasgow, Scotland. SVCBAK is a line of CBA mouse kidney cells transformed with SV40 virus. L/R cells are a TNF-resistant subline of L cells (Matthews and Watkins, 1978).

*Cytotoxicity assay.*—In Sterilin M29ARTL microplates, 75 $\mu$l of test substance (either leucocyte suspension or supernatant, or TNF serum) or control substance (MEM20 or control serum) was added to 75 $\mu$l of the target-cell suspension ($10^5$/ml). After incubation for 3 or 4 days at 37°C in 95% air, 5% $CO_2$, the target cells were washed $\times 2$ with PBS, fixed with methanol and stained with Giemsa. The centre of each well was located at low magnification and the cells in one high-power field in the centre of the plate were counted. The % cytotoxicity was calculated from the formula $100(a-b)/a$ where $a$ and $b$ are respectively the mean number of cells in 6–8 wells with control or test substance. For tests with leucocytes or TNF, MEM20 was used as the control, and for tests with leucocyte supernatants, MEM20 incubated at 37°C for the same length of time as the supernatant was usually used. However, as this gave identical results to unincubated MEM20, the latter was used in later experiments.

*Identification of monocytes.*—Monocytes were identified in fixed and Giemsa-stained preparations on the basis of morphology, and on the capacity to ingest China ink (de Halleux *et al.*, 1973).

*Supernatant fractionation.*—Ammonium sulphate precipitation was performed as described previously (Matthews and Watkins, 1978). Downward-flow gel filtration in sterile PBS was performed with a $1{\cdot}5 \times 38$ cm Ultrogel AcA44 column at a flow rate of 1·9 ml/h, and 1·0 ml fractions were collected.

For polyacrylamide electrophoresis in 7% gel rods (Tombs and Akroyd, 1967) a discontinuous buffer system, pH 8·6, was employed (Poulik, 1957) with a constant current of 2 mA/tube. After the bromophenol blue marker had just reached the end of the gel, electrophoresis was stopped, gels were removed from their tubes, frozen and chopped into 12 aliquots. The slices were disrupted with forceps, suspended in 1 ml PBS and dialysed overnight against PBS before testing the supernatant. All fractions were sterilized with a 0·2 $\mu$m filter prior to testing.

*Phagocytosis.*—Monocyte-enriched fractions obtained by plastic adherence were incubated at 37°C with 150 $\mu$l suspensions (0·25% v/v) of antibody-coated sheep erythrocytes (EA) or aged sheep erythrocytes, or with a suspension ($2 \times 10^7$/ml) of formolized *Candida albicans*. EA were made by incubation of 1% (v/v) E with an equal volume of a 1/100 dilution of Rose-Waaler reagent (Wellcome Reagents).

### RESULTS

Previously it was noted that TNF was cytotoxic *in vitro* to a limited number of cell lines. In studies on "spontaneous" cytotoxicity by mononuclear cells from rabbit blood, a similar limited specificity was noted. Fig. 1 shows the results of an experiment in which blood mononuclear

FIG. 1.—Comparison of TNF (▲) and mononuclear cells (△) from the blood of rabbits for cytotoxicity against a range of cell lines in a 3-day assay. Effector: target cell ratio =40:1.

cells from 4 unimmunized rabbits were compared with TNF for cytotoxicity in a 3-day assay against a range of cell lines. Those cell lines most susceptible to TNF were also those most susceptible to "spontaneous" mononuclear-cell cytotoxicity. Furthermore, the L/R sub-line of L cells, selected for resistance to TNF, was only weakly sensitive to mononuclear-cell cytotoxicity. (The Chang and L/R lines are not intrinsically resistant to cytolysis, as both cell lines are highly susceptible to antibody-dependent cell-mediated cytotoxicity.)

The mononuclear-cell preparation comprised mostly lymphocytes with 10–20% monocytes. Granulocyte contamination was usually less than 2%. To determine the cell type responsible for cytotoxicity, the cells were separated into a plastic-adherent fraction (mostly monocytes) and a non-adherent fraction (mostly small lymphocytes). The bulk of the cytotoxic activity was expressed by the monocyte-enriched, plastic-adherent fraction (Table I). Depletion of monocytes by adherence to a cotton-wool column also markedly reduced cytotoxicity (Table I).

To investigate whether "spontaneous" cytotoxicity is due to the release from cells of a TNF-like factor, the supernatants from 3-day cultures of rabbit mononuclear cells were tested for cytotoxicity against L cells (Table II). Supernatants were cytotoxic to L cells, irrespective of whether the mononuclear-cell culture medium was supplemented with foetal calf or autologous serum. Although some cytotoxicity was detectable at a supernatant dilution of 1/400, TNF serum is at least 10× more potent. The specificity of the supernatant (Table III) was similar to the parent cells and to TNF (compare Fig. 1 and Table III). Unique amongst the cell lines tested, SVCBAK exhibits a widely variable sensitivity to TNF when tested on different

TABLE I.—*Effect of purification by adherence to plastic or cotton wool on mononuclear-cell cytotoxicity against L cells*

| Mononuclear cell population | % Monocytes | Effector : target ratio | No. of L cells* ±s.d. | % Cytotoxicity |
|---|---|---|---|---|
| Control medium | | | 139±16 | |
| Non-fractionated | 12 | 50 | 62±14 | 55·4 |
| | | 12·5 | 67±12 | 51·8 |
| | | 3·1 | 95±21 | 31·7 |
| Plastic-adherent | 75 | 8·3 | 75±24 | 46·0 |
| Non-adherent to plastic | <1 | 50 | 80±15 | 42·4 |
| | | 12·5 | 106±26 | 23·7 |
| | | 3·1 | 125±13 | 10·1 |
| Non-adherent to cotton wool | <1 | 50 | 100±9 | 28·1 |
| | | 12·5 | 135±14 | 2·9 |
| | | 3·1 | 118±19 | 15·1 |

* Counted after 3 days.

TABLE II.—*Cytotoxicity against L cells by supernatants of mononuclear cells cultured in the presence of foetal calf or autologous serum*

| | Medium supplement (serum) | Supernatant dilution | No. of L cells† ±s.d. | % Cytotoxicity |
|---|---|---|---|---|
| Control medium | | | 139±28 | |
| Supernatant* | Foetal calf | 1/4 | 12±5 | 91·4 |
| | | 1/40 | 51±13 | 63·3 |
| | | 1/400 | 108±20 | 22·3 |
| Supernatant* | Autologous | 1/4 | 6±5 | 95·7 |
| | | 1/40 | 78±10 | 43·9 |
| | | 1/400 | 150±25 | −7·9 |

* Supernatants of rabbit blood mononuclear cells ($5 \times 10^6$/ml) cultured for 3 days.
† Counted 3 days after the addition of the supernatants. Both supernatants were diluted with medium containing foetal calf serum.

occasions (compare Fig. 1 and Table III). Although the reason for this variability is unknown, SVCBAK cells show an exactly parallel variation in susceptibility to the mononuclear cell supernatant.

TABLE III.—*Comparison of the cytotoxic specificity of the mononuclear-cell supernatant with TNF*

| Target cells | Additive | No. of cells* ±s.d. | % Cytotoxicity |
|---|---|---|---|
| L | Control medium | 59±9 | |
| | TNF/160 | 0·0±0 | 100·0 |
| | Sup†/2 | 0·3±1 | 98·8 |
| L/R | Control medium | 136±16 | |
| | TNF/160 | 122±22 | 10·3 |
| | Sup/2 | 132±9 | 2·9 |
| Chang | Control medium | 47±11 | |
| | TNF/160 | 53±12 | −12·8 |
| | Sup/2 | 38±7 | 19·1 |
| SVCBAK | Control Medium | 66±15 | |
| | TNF/160 | 7±2 | 89·4 |
| | Sup/2 | 9±3 | 86·4 |

* Counted after 4 days.
† Sup = mononuclear-cell supernatant.

From Table IV it can be seen that detectable amounts of the cytotoxic factor are released from mononuclear cells after as little as 3 h in culture at 37°C, and maximal amounts are released by 7 h. Other experiments using incubation periods of up to 72 h, have shown no further increase in activity. No cytotoxic activity was found in the supernatant after culture at 4°C or at room temperature, or after disruption of the cells by freezing and thawing 8 times. The cytotoxic factor was released by the plastic-adherent, monocyte-enriched fraction, and not by the non-adherent, lymphocyte-enriched fraction (Table V). In a separate experiment it was shown that the supernatant of a granulocyte-enriched fraction was 7·5× less cytotoxic to L cells than the supernatant of the monocyte fraction from

TABLE IV.—*Comparison of supernatants of mononuclear cells cultured for different times for cytotoxicity against L cells*

| | Time (h) | Supernatant dilution | No. of cells* ±s.d. | % Cytotoxicity |
|---|---|---|---|---|
| Control medium | | | 93±13 | |
| Supernatant† | 1 | 1/8 | 83±8 | 10·8 |
| | | 1/80 | 91±16 | 2·2 |
| | | 1/8 | 36±5 | 61·3 |
| | | 1/80 | 76±11 | 18·3 |
| | 7 | 1/8 | 14±4 | 84·9 |
| | | 1/80 | 54±12 | 41·9 |
| | 22 | 1/8 | 15±3 | 83·9 |
| | | 1/80 | 50±4 | 46·2 |

* Counted 3 days after addition of supernatant.
† From cultures of blood mononuclear cells at $5 \times 10^6$/ml.

TABLE V.—*Comparison of 3-day supernatants of plastic-adherent or non-adherent mononuclear cells for cytotoxicity against L cells*

| | No. of effector cells/ml (×10⁶) | % Monocytes | % Cytotoxicity* of supernatant at 1/4 | 1/40 | 1/400 |
|---|---|---|---|---|---|
| Adherent | 0·2 | 75 | 67·0 | 17·2 | 4·5 |
| Non-Adherent | 2·3 | <2 | 5·3 | 1·7 | −10·1 |

* 3-day assay.

the same blood sample. There was neither an earlier nor increased production of the factor by monocytes phagocytosing aged sheep erythrocytes, antibody-coated erythrocytes or killed *Candida albicans*.

Over 90% of the cytotoxicity activity of both TNF and the monocyte supernatant was found in the pellet after precipitation in 50% saturated ammonium sulphate solution. On gel filtration using Ultrogel AcA44 (Fig. 2) both TNF and the monocyte factor appeared heterogeneous, with the bulk of the material eluted in the range 30,000–50,000 u. On polyacrylamide electrophoresis, both substances migrated just behind albumin (Fractions 10 and 11) with TNF being eluted from Fractions 8 and 9 and the monocyte factor from Fraction 9 (Table VI).

FIG. 2.—Ultrogel AcA44 gel-filtration profiles of (a) TNF serum and (b) monocyte supernatant. The fractions were tested for cytotoxicity against L cells at dilution of (a) 1/100 (b) neat. The supernatant was from a 24 h culture containing 97% monocytes. The arrows indicate the elution volumes of IgG, albumin (Alb), ovalbumin (Ova) and soy-bean trypsin inhibitor (TI).

### DISCUSSION

The cytotoxic specificity of rabbit TNF against a range of cell lines was similar to

TABLE VI.—*Cytotoxicity against L cells by fractions of TNF serum or monocyte supernatant obtained by polyacrylamide-gel electrophoresis*

| | TNF serum | | Monocyte supernatant* | |
|---|---|---|---|---|
| Fraction no. | No. of L cells† ±s.d. | % Cytotoxicity | No. of L cells† ±s.d. | % Cytotoxicity |
| Control | 53±5 | | 77±12 | |
| 1 | 50±8 | 5·7 | 78±11 | −1·3 |
| 2 | 54±12 | −1·9 | 76±7 | 1·3 |
| 3 | 57±8 | −7·5 | 79±12 | −2·6 |
| 4 | 52±7 | 1·8 | 80±12 | −3·9 |
| 5 | 54±2 | −1·8 | 78±13 | −1·3 |
| 6 | 54±8 | −1·8 | 77±11 | 0·0 |
| 7 | 51±7 | 3·8 | 76±8 | 1·3 |
| 8 | 35±5 | 34·0 | 78±13 | −1·3 |
| 9 | 25±6 | 52·8 | 30±5 | 61·0 |
| 10 | 56±5 | −5·7 | 73±9 | 5·2 |
| 11 | 51±7 | 3·8 | 80±10 | −3·9 |
| 12 | 49±4 | 7·5 | 73±8 | 5·2 |

* From a 24 h supernatant of a culture containing 95% monocytes.
† 4-day assay. Fractions were tested at 1/10 dilution for TNF and neat for the monocyte supernatant.

that exhibited by mononuclear cells from the blood of healthy unimmunized rabbits. A cytotoxic factor could be detected in the supernatant of mononuclear cells incubated for as little as 3 h *in vitro* at 37°C. This factor had the same specificity as the parent cells and TNF. In addition, like TNF, it precipitated in 50% saturated ammonium sulphate solution and was eluted on gel filtration with the same mol. wt. range. It appears, therefore, that TNF and the cytotoxic factor are closely related, if not identical. As rabbit TNF is poorly immunogenic in sheep and guinea-pigs it has not yet been possible to test for antigenic cross-reactivity between TNF and the cytotoxic factor.

The cytotoxic factor was produced by plastic-adherent cells, probably monocytes, and this is consistent with the suggestion of Old (1976), based on indirect evidence, that TNF is produced by cells of the macrophage series. Reed and Lucas (1975) noted that supernatants of human or rat macrophage cultures were cytotoxic to some cell lines, and the temporal production of the factors and their molecular weights were similar to those reported here. Thus, TNF or closely related factors appear to be produced normally by macrophages. In animals primed with agents such as BCG, which induce macrophage hyperplasia, endotoxin-induced lysis of macrophages would lead to release of large amounts of TNF into the circulation. It cannot be excluded that the TNF-like factor described here may be released from monocytes by cell damage caused by minute amounts of endotoxin in the culture medium.

There are striking similarities between TNF and rabbit interferon. Both can be produced *in vivo*, 2 h after challenge with endotoxin. *In vitro*, interferon and the TNF-like factor are released from mononuclear phagocytes after short incubation periods of 37°C, but not at 4°C, nor on cell disruption (Smith and Wagner, 1967). In terms of physicochemical properties, there is also a close resemblance between rabbit TNF and interferon. Human and mouse interferons can inhibit the growth of some cell lines (see Gresser, 1977), whilst human interferon can be cytotoxic (Kuwata *et al.*, 1976). Like its anti-viral action, the growth-inhibitory effects of interferon seem to be species specific. In contrast, rabbit TNF is cytotoxic to certain mouse cell lines as well as a rabbit line, and we have also recently observed a growth-inhibitory effect on a human melanoma cell line.

I thank Mrs M. L. Neale for excellent technical assistance.

REFERENCES

CARSWELL, E. A., OLD, L. J., KASSEL, R. L., GREEN, S., FIORE, N. & WILLIAMSON, B. (1975) An endotoxin-induced serum factor that causes necrosis of tumours. *Proc. Natl. Acad. Sci., U.S.A.*, **72**, 3666.

DE HALLEUX, F., TAPER, M. S. & DECKERS, C. (1973) A simple procedure for identification of macrophages in peritoneal exudates. *Br. J. Exp. Path.*, **54**, 352.

GRESSER, I. (1977) On the varied biologic effects of interferon. *Cell. Immunol.*, **34**, 406.

KUWATA, T., FUSE, A. & MORINAGA, N. (1976) Effects of interferon on cell and virus growth in transformed human cell lines. *J. Gen. Virol.*, **33**, 7.

MATTHEWS, N. & WATKINS, J. F. (1978) Tumour necrosis factor from the rabbit. I. Mode of action, specificity and physicochemical properties. *Br. J Cancer*, **38**, 302.

OLD, L. J. (1976) Tumor necrosis factor. *Clin. Bull.* **6**, 118.

POULIK, M. D. (1957) Starch gel electrophoresis in a discontinuous system of buffers. *Nature*, **180**, 1477.

REED, W. P. & LUCAS, Z. J. (1975) Cytotoxic activity of lymphocytes. V. Role of soluble toxin in macrophage-inhibited cultures of tumor cells. *J. Immunol.*, **115**, 395.

SMITH, T. J. & WAGNER, R. R. (1967) Rabbit macrophage interferons. I. Conditions for biosynthesis by virus-infected and uninfected cells. *J. Exp. Med.*, **125**, 559.

TOMBS, M. P. & AKROYD, P. (1967) Acrylamide gel electrophoresis. *Shandon Instrument Applications*, 18.